# Correlation of West Nile Virus Incidence in Donated Blood with West Nile Neuroinvasive Disease Rates, United States, 2010–2012

**DOI:** 10.3201/eid2302.161058

**Published:** 2017-02

**Authors:** Edouard Betsem, Zhanna Kaidarova, Susan L. Stramer, Beth Shaz, Merlyn Sayers, German LeParc, Brian Custer, Michael P. Busch, Edward L. Murphy

**Affiliations:** University of Yaounde 1 Faculty of Medicine and Biomedical Sciences, Yaounde, Cameroon (E. Betsem);; Agence de Médecine Préventive, Bobo-Dioulasso, Burkina Faso (E. Betsem);; Blood Systems Research Institute, San Francisco, California, USA (E. Betsem, Z. Kaidarova, B. Custer, M.P. Busch, E.L. Murphy);; American Red Cross, Gaithersburg, Maryland, USA (S.L. Stramer);; New York Blood Center, New York, New York, USA (B. Shaz);; Carter BloodCare, Bedford, Texas, USA (M. Sayers);; University of Texas Southwestern Medical Center, Dallas, Texas, USA (M. Sayers);; OneBlood, St. Petersburg, Florida, USA (G. LeParc);; University of California, San Francisco (B. Custer, M.P. Busch, E.L. Murphy)

**Keywords:** West Nile virus, WNV, blood donors, incidence, arbovirus, neuroinvasive disease, nucleic acid testing, viruses, United States, surveillance, demographic factors, seasonal variation, geographic variation, zoonoses, vector-borne infections, antibodies

## Abstract

Over the past decade, West Nile virus (WNV) has spread across the United States. We aggregated blood donor data from 2010–2012 and then calculated the incidence of WNV RNA–positive donations and compared the incidence with neuroinvasive disease (NID) case data from the ArboNET surveillance system. Of 10,107,853 donations, 640 were confirmed positive. The seasonal WNV incidence rate per 100,000 persons was 33.4 (95% CI 22–45) in 2010, 25.7 (95% CI 15–34) in 2011, and 119.9 (95% CI 98–141) in 2012. NID to blood donor ratios were 1 in 164 (95% CI 152–178) in 2010, 1 in 158 (95% CI 145–174) in 2011, and 1 in 131 (95% CI 127–136) in 2012. We updated estimates of the ratio of NID to WNV infection rates, demonstrating stable disease penetrance over the study period. Blood donor WNV RNA screening is a valuable public health tool for WNV surveillance.

West Nile virus (WNV), a mosquitoborne flavivirus, was first isolated in 1937 from a patient in Uganda (*1*). The virus was introduced into the United States in 1999, resulting in a focal epidemic that year in New York, New York. WNV then made a westward migration across the United States, becoming an endemic public health problem that is monitored through the Centers for Disease Control and Prevention (CDC; Atlanta, GA, USA) ArboNET surveillance system (*2*). Although most WNV infections are asymptomatic, they can cause a syndrome of fever and myalgia in a minority of cases and can also cause neuroinvasive disease (NID) manifesting as meningitis, encephalitis, or acute flaccid paralysis (*2*–*6*). An estimated 20%–25% of infected persons become symptomatic (5). During 1999–2010, an estimated 2–4 million WNV infections, 0.4–1 million cases of febrile illness, and 13,000 reported cases of NID occurred in the United States (*7*). US public health authorities received >39,000 reports of WNV infections during 1999–2013 (*8*). Early data from 1999 indicated that NID develops in <1 in 150 WNV-infected persons (*5*). However, based on 2003 data, this estimate was revised to 1 in 256 based on a correlation of NID case-report data with total WNV infection projections derived from blood donor WNV testing data (*9*).

The risk of transmitting WNV through blood transfusion was anticipated by CDC investigators and estimated to be as high as 2.7 transmissions/10,000 transfused blood units during the peak of the 1999 outbreak (*10*); however, confirmed transfusion-transmitted infections were not reported until 2002 (*11*,*12*). After these reports, the US Food and Drug Administration, CDC, US blood centers, and test manufacturers quickly collaborated to develop and implement, beginning in summer 2003, routine nucleic acid testing (NAT) to screen blood donors for WNV RNA (*13*,*14*).

Predictions of the scale of annual outbreaks are not reliable (*7*). Environmental temperature and precipitation data may serve to estimate when WNV transmission rates in mosquitoes and birds will exceed specific thresholds, such that the probability of transmission to humans would be predicted to occur (*15*,*16*). Blood center data are a useful adjunct because testing laboratories monitor the number of WNV NAT–positive donations in near real time to determine if they should convert from NAT of minipools (MP-NAT) to more sensitive NAT of individual donations (ID-NAT) (*17*). In addition, WNV NAT–positive blood donations and NID cases are reported to CDC by state and territorial health laboratories via the ArboNET surveillance system. These reports showed that, after relatively low numbers of WNV outbreaks during 2004–2011, the WNV incidence rate in 2012 was one of the highest reported. The NID-associated death rate in 2012 was 9.9%, and the number of deaths (286) is the highest annual number reported to CDC; of the 286 deaths, 55.5% (159) were reported from 5 states: 89 (31%) from Texas, 20 (7%) from California, 16 (5.6%) in Louisiana, and 17 (6%) each from Michigan and Oklahoma (*18*).

Blood donors represent a readily accessible sample of the US population that is systematically screened for incident WNV infections. This screening provides an approximation of the magnitude of the WNV epidemic each year, and this estimate complements data reported to ArboNET on cases of symptomatic WNV-associated disease. Busch et al. (*9*) previously estimated national and state-specific WNV infection rates in 2003 from the number and frequency of WNV NAT–positive blood donations and used those rates to provide statewide projections, which were then correlated with NID case rates. In this study, we used a larger dataset of WNV NAT–positive blood donations to model the US population incidence of WNV during 2010–2012. We also provide updated ratios of the estimated number of WNV infections to NID cases.

## Methods

### Study Population

Blood donor data were extracted from operational blood center databases and provided without personal identifiers. The total number of donations and the number of NAT-confirmed WNV-positive donations were categorized by donor age, sex, and state of residence. Data were collected over the 5-month epidemic period from June 1 to October 31 during 2010– 2012. During 2003–2012, only 5 WNV NAT-positive blood donations were reported outside the months (June–October) that we included in this analysis: 4 occurred in November 2012 and 1 in April 2010 (*19*). Data for this study were obtained from the American Red Cross (Washington, DC, USA), which collects blood in 44 US states and Washington, DC; Blood Systems, Inc. (Scottsdale, AZ, USA), whose centers collect mostly in the Southwest, the Central Plains, and parts of California; the New York Blood Center (New York, NY, USA), which collects in New York and New Jersey; Carter Blood Care (Bedford, TX, USA), which collects in northern Texas; and OneBlood (Tampa, FL, USA), which collects blood throughout Florida and southern Georgia. Altogether, the current dataset is estimated to capture ≈60% of US blood donations. Blood centers provided count data stratified by age, date of donation, sex, and geographic location, and Blood Systems, Inc. provided person-specific data on all donors to enable risk factor analysis. This analysis did not constitute human subjects research because only existing data without personal identifiers were available to the investigators.

### Blood Donor Screening

During the time of the study, blood donations from Blood Systems, Inc., the New York Blood Center, and Carter Blood Care were tested at Creative Testing Solutions laboratories (Tempe, AZ, USA) for WNV RNA by minipool NAT (pools of 16) and ID-NAT for resolution of donations within reactive pools, both using transcription-mediated amplification (TMA) (Hologic, San Diego, CA, USA; Grifols Diagnostics, Emeryville, CA, USA). The American Red Cross performed WNV NAT at its National Testing Laboratories (Stockbridge, GA, USA) using the same TMA assay. OneBlood used MP-NAT (pools of 6) based upon a PCR technique (Roche Molecular Systems, Branchburg, NJ, USA) or MP-NAT (pools of 16; Hologic/Grifols); resolution of donations within reactive pools was done using ID-NAT. Results from MP-NAT testing are monitored to determine if epidemic activity requires the triggering of more sensitive ID-NAT testing in geographic areas experiencing outbreaks (*17*,*20*–*25*). The trigger for converting from MP-NAT to ID-NAT in response to ongoing WNV activity was 1–2 reactive blood donations; for the TMA system, this was restricted to those reactive donations having a high ID-NAT signal (*17*,*25*,*26*).

NAT-reactive blood donations can represent a WNV-infected donor or a falsely reactive test result; thus, results must be confirmed by repeating NAT on an independent sample, by demonstrating donor seroconversion, or both (*17*,*25*). In our study, we included all ID-NAT–confirmed positive donations whether they were initially screened by MP-NAT or ID-NAT.

### Statistical Analysis

Using only Blood Systems, Inc., data for confirmed WNV-positive and negative donations, we performed a univariate analysis of WNV NAT reactivity by donation year and donor sex and age group. We compared categorical variables by using the χ^2^ test; age as a continuous variable was compared using the Student *t*-test. We estimated odd ratios in a logistic regression model that included adjustment for age group, sex, region of residence, season, and month.

Most WNV RNA–positive persons who donate blood are asymptomatic or in the presymptomatic stage of infection; thus, we calculated the seasonal incidence of WNV on the overall dataset, assuming independence between blood donation and WNV infection (*25*,*27*,*28*). For this analysis, we used WNV RNA detection periods (i.e., number of days between first testing positive and testing negative) of ≈10.7 days by MP-NAT and ≈19.6 days by ID-NAT (*29*). These estimates were adapted from the method of Busch et al. (*9*), using data from Kleinman et al. (*30*). We did not have access to data on whether donations were screened by MP-NAT or ID-NAT. Given that roughly equal proportions of yield donations were derived from MP-NAT and ID-NAT screening, we used an average window of 15.1 days, assuming a 50% mixture of ID-NAT and MP-NAT screening donations during the epidemic period. We then multiplied the total donations screened for WNV RNA by 15.1 days to derive person-time for the denominator in incidence calculations; the number of corresponding NAT yield donations was used as the numerator (*9*).

We derived the monthly WNV incidence in each state from June through October by multiplying the number of NAT-positive donations for each month by the number of days in each month and dividing by the average period of time during which RNA is detectable (*9*). We calculated state-specific seasonal WNV incidence by adding the 5 monthly WNV incidence estimates for each year. An estimation of the number of WNV infections in each state was calculated by multiplying each state-specific seasonal WNV incidence by the corresponding population estimate obtained from the US Census Bureau (*31*). We then obtained a national seasonal estimate by summing over the estimates for participant states. An overall seasonal incidence for the 3 years was calculated weighted on the general population for each year. CIs were obtained assuming a Poisson distribution for NAT-positive donations.

We obtained the ratio of WNV infections to reported NID cases by state by dividing the estimated number of infections in the general population by state by the number of NID cases reported to ArboNET. This estimation was repeated for each year. We obtained CIs by applying Taylor series expansion (*32*). Analysis of correlation between WNV incidence and reported NID through ArboNet surveillance was done using a linear regression. Correlations and summarizations were expressed using R^2^. All statistical analyses were performed using Stata 12.1 (StataCorp LP, College Station, Texas, USA). We prepared graphical (maps) displays of results using ArcGIS version 9.3.1 (ESRI, Redlands, CA, USA). We did not conduct a county-level analysis.

## Results

### Study Population and Demographic Predictors

A total of 10,107,853 blood donations collected during June–October in 2010–2012 were included in this study: total donations for 2010 were 3,470,405, total donations for 2011 were 3,360,443, and total donations for 2012 were 3,277,005. All donors included in the analysis were US residents; 20% lived in Western states, 27% in the Midwestern states, 32% in Southern states, and 21% in Northeastern states. Data from Blood Systems, Inc., indicated that blood donors ranged in age from 16 to 98 years (median age 45 years), and men accounted for 53% of donations.

**Figure 1:**
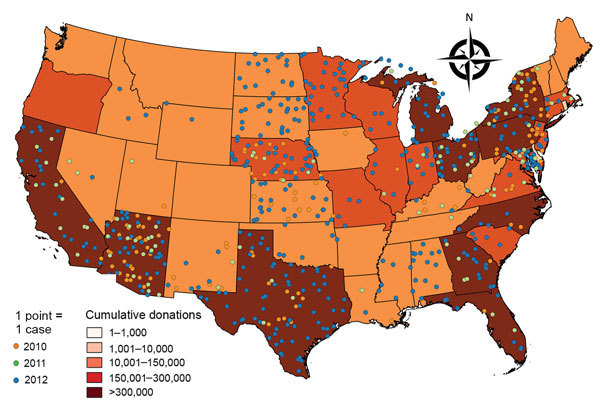
Geographic distribution of blood donations confirmed positive for West Nile virus (WNV) RNA, United States, June–October 2010–2012. The 640 confirmed WNV DNA–positive donations are represented by dots. Shading indicates cumulative number of donations for 2010–2012, by state, for catchment areas of >1,000 donations.

Overall, 640 donations were WNV NAT positive (Figure 1). WNV RNA–positive blood donations clustered according to WNV epidemic activity and the catchment areas of the participating blood collection networks. Apparent clustering was observed in Southwest, Central, and Northeast states in 2010 and in Southwest and Northeast states in 2011; the pattern was much more dispersed in 2012, involving the North Central, Southwest, and Northeast states.

The frequency of WNV RNA was 63% higher among male than female donors and 122% higher among white than nonwhite donors. Donors from the Midwest had higher rates of WNV infection. Higher rates of NAT-positive donations were observed in 2012 versus 2010, but rates in 2011 were significantly lower than those in 2010 (Table).

**Table:** Demographic characteristics of WNV RNA–positive persons who donated blood during WNV seasons, United States, 2010–2012*

Characteristic	No. WNV NAT–positive/no. total (%)	Crude OR (95% CI)	Adjusted OR (95% CI)
Age group			
<20	13/154,874 (0.01)	Referent	Referent
21–29	17/172,947 (0.01)	1.17 (0.56–2.41)	0.96 (0.42–2.20)
30–39	27/164,611 (0.02)	1.95 (1.01–3.78)	1.63 (0.74–3.58)
40–49	27/205,907 (0.01)	1.56 (0.80–3.02)	1.31 (0.60–2.86)
>50	68/498,320 (0.01)	1.62 (0.89–2.94)	1.21 (0.58–2.51)
Sex			
F	54/566,850 (0.01)	Referent	Referent
M	98/629,809 (0.02)	1.63 (1.17–2.27)	1.59 (1.13–2.22)
Race			
Nonwhite	20/280,246 (0.01)	Referent	Referent
White	131/826,704 (0.02)	2.22 (1.38–3.55)	1.75 (1.06–2.89)
Region of residence			
Northeast	0/900	Omitted	Omitted
West	75/765,873 (0.01)	Referent	Referent
Midwest	44/141,507 (0.03)	3.17 (2.18–4.60)	2.54 (1.73–3.73)
South	33/288,379 (0.01)	1.16 (0.77–1.75)	1.11 (0.73–1.70)
Year			
2010	44/413,840 (0.01)	Referent	Referent
2011	18/399,980	0.42 (0.24–0.73)	0.43 (0.24–0.74)
2012	90/382,839 (0.02)	2.21 (1.54–3.17)	2.19 (1.52–3.15)
Month			
June	5/240,481	Referent	Referent
July	28/238,208 (0.01)	5.65 (2.18–14.64)	5.44 (2.09–14.13)
August	76/241,994 (0.03)	15.10 (6.11–37.34)	14.62 (5.90–36.18)
September	39/236,036 (0.02)	7.94 (3.13–20.16)	8.34 (3.28–21.20)
October	4/239,940	0.80 (0.21–2.98)	0.83 (0.22–3.11)

*Data are from Blood Systems, Inc. (Scottsdale, AZ, USA); a total of 1,196,659 donations were included. OR, odds ratio; WNV, West Nile virus.

### WNV Seasonal Incidence Analysis

**Figure 2:**
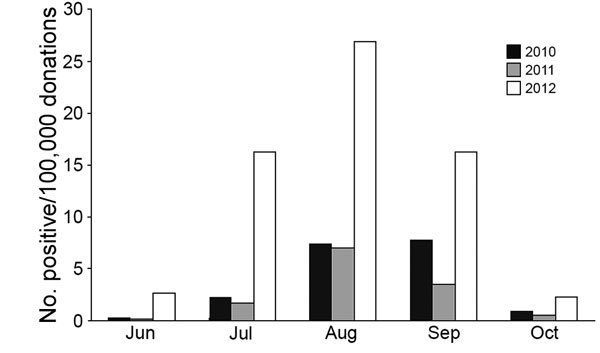
Rate of West Nile virus (WNV)–positive blood donations by month, United States, June–October 2010–2012. Overall, 640 WNV-positive donations were confirmed by nucleic acid testing in 10,107,853 screened donations. Positive donations collected during the months of January–May (1 in April 2010) and November–December (3 in November 2012) are not shown.

Seasonal rates were 3.7 cases/100,000 donations (≈1 in 26,700) in 2010, 2.6 cases/100,000 donations in 2011 (≈1 in 38,200), and 12.9 cases/100,000 donations (≈1 in 7,800) in 2012. Over the 3 years, WNV activity was highest in August and September, as evidenced by NAT-positive rates; in 2010, rates peaked in September (7.7 cases/100,000 donations), and in 2011, rates peaked in August (7.0 cases/100,000 donations) (Figure 2). In 2012, NAT-positive rates peaked in August (26.9 cases/100,000 donations), but activity was high from July (16.2 cases/100,000 donations) through September (16.2 cases/100,000 donations).

**Figure 3:**
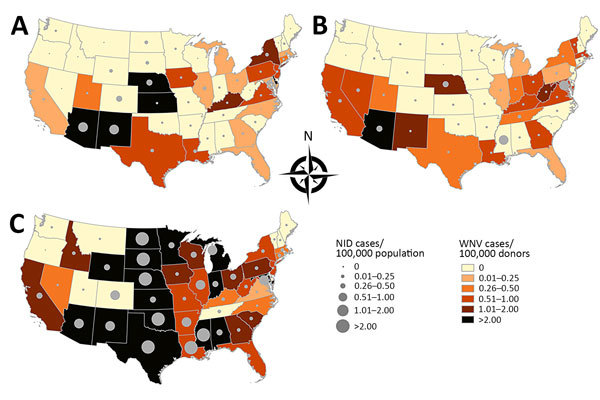
Incidence of West Nile virus (WNV) infection cases and neuroinvasive disease (NID) cases by state, United States, 2010–2012. Dots indicate attack rates for NID cases reported to the Centers for Disease Control and Prevention in 2010 (A), 2011 (B), and 2012 (C).

Diverse geographic incidence patterns were observed over the 3 years (Figure 3). In 2010 and 2011, the states with the highest activity were Arizona, New Mexico, Nebraska, and Kansas; the incidence in 2011 was lower than that in 2010. In 2012, the epidemic grew in scale and expanded to Texas and North Central states, including South Dakota, North Dakota, Minnesota, and Wyoming. High infection incidence was also observed in Alabama and Mississippi.

Overall seasonal WNV incidence estimates were 33.4 cases/100,000 persons in 2010 (Technical Appendix Table 1) and 24.7 cases/100,000 persons in 2011 (Technical Appendix Table 2). The estimated incidence for 2012 was 119.9 cases/100,000 persons. Among states, incidence ranged from 12.9 cases/100,000 persons in Virginia to 766.9 and 1,465.4 cases/100,000 persons in North and South Dakota, respectively (Technical Appendix Table 3).

**Figure 4:**
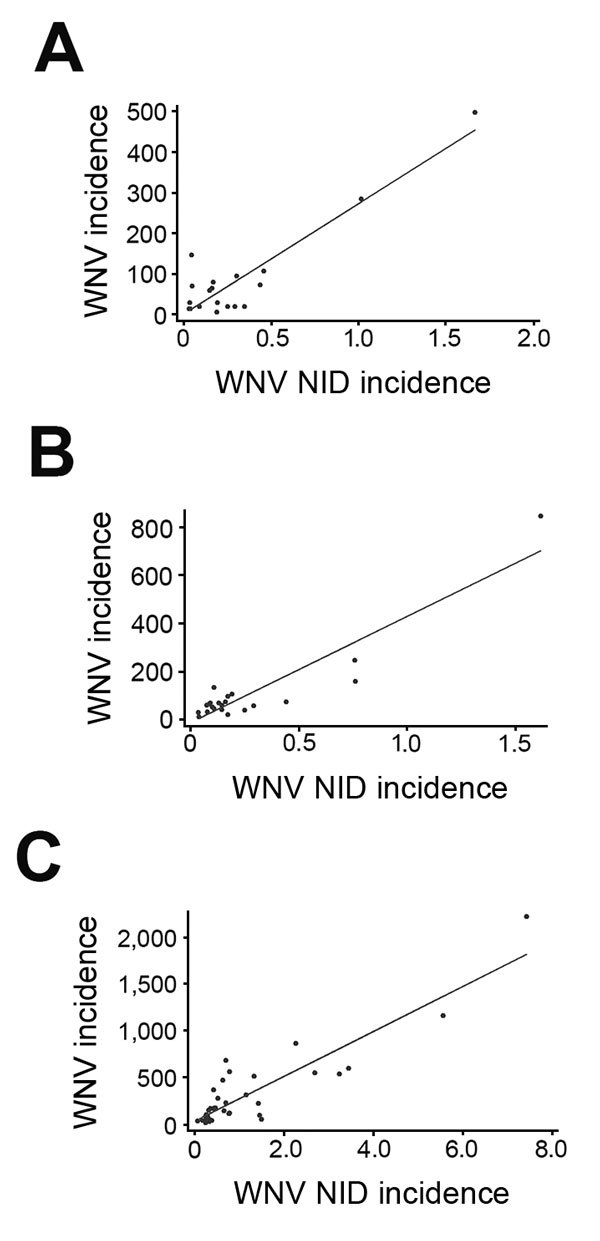
Projected West Nile virus (WNV) incidence (per 100,000 population) versus neuroinvasive disease (NID) case rates (per 100,000 population) by state, United States, 2010–2012. Each dot within each panel represents 1 state. A) Data for 2010. Data from 30 states were excluded: 11 states with no donors testing positive by nucleic acid testing (NAT) and no NID cases; 17 states with 1–26 NID cases but no NAT-positive donors; and 2 states with 1 and 2 NAT-positive donors, respectively, but no reported NID cases (R^2^ = 0.83). B) Data for 2011. Data from 27 states were excluded: 8 states with neither NAT testing yield nor NID cases and 19 states with 1–31 NID cases but no NAT yield (R^2^ = 0.83). C) Data for 2012. Data from 15 states were excluded: 3 states with neither NAT testing yield nor NID cases and 12 states with 1–62 neuroinvasive cases but no NAT yield (R^2^ = 0.79).

National and state-specific variability in projected WNV infections generally paralleled NID rates reported to ArboNET (Figure 3). In 2010, 2011, and 2012, 629, 486, and 2,872 NID cases, respectively, were reported. Cumulative national estimates of WNV cases were 103,450 cases in 2010 and 76,975 cases in 2011, and the ratio of NID cases to WNV infections was 1 to 164 (95% CI 152–178) in 2010 and 1 to 158 (95% CI 145–174) in 2011 (Figure 4; Technical Appendix Tables 1, 2). In 2012, an estimated 376,612 WNV infections occurred, and the ratio of NID cases to WNV infections was 1 to 131 (95% CI 126–136) (Figure 4; Technical Appendix Table 3). Over the 3 years of the study, the weighted ratio of NID cases to WNV infections in the general population was 1 to 141 (95% CI 118–164). In addition, during 2010–2012, projected incidence correlated with NID case frequencies (R^2^ value of 0.83 in 2010, 0.83 in 2011, and 0.79 in 2012) (Figure 4).

## Discussion

Our findings, which extended previous findings (*9*), highlight the value of using WNV NAT–positive blood donation data to model population incidence in the United States. Our period of data collection covered 3 years, including the large WNV epidemic in 2012, enabling us to demonstrate seasonal and geographic variation in incidence. Using a large geographic catchment area and multiple years of data, we were able to provide updated estimates of the ratio of NID cases to WNV infections, demonstrating stable disease penetrance over the study period and that our estimate (1:141) is closer to that reported for the year 1999 (1:140) (*5*) than that reported for the year 2003 (1:256) (*9*).

Our analysis of demographic factors shows seasonal and geographic variations of WNV infection rates in blood donors. This study’s incidence estimate of 12.9 infections/100,000 blood donations in 2012 is the same as that reported by Francis et al. (*33*) for the 2010 outbreak in New York but lower than the 20 and 27 cases/100,000 persons reported for national data in 2003 (*9*,*21*). Kleinman et al. (*20*) reported a higher rate of 35 cases/100,000 blood donations for the 2003 epidemic for a specific group of blood centers. Using American Red Cross data and a well-defined confirmatory algorithm (similar to the method in our study), Stramer et al. (*22*) reported infection rates of 14.9 and 4.4 cases/100,000 blood donations in 2003 and 2004, respectively; these rates are higher than those we estimated for 2010 and 2011 (3.7 and 2.6 cases/100,000 donations, respectively), but rates in 2003 and 2012 appeared comparable. Projected WNV incidence in the general population and NID case frequency decreased slightly from 2010 to 2011 and then spiked upward in 2012 during an outbreak that spread to the Midwest with high incidence rates (Technical Appendix Table 3); this pattern was similar to that observed during the 2003 epidemic (http://www.cdc.gov/westnile/resources/pdfs/data/2003stateincidencemap.pdf). These results are in agreement with our data showing that the highest projected incidence generally correlated with NID case frequencies (Figure 4).

Patterns of WNV activity vary from year to year, exhibiting temporal and geographic variations of incidence, as shown by our data in the blood donor pool and corresponding projections in general population incidence. Fourteen years after their first appearance in the United States, WNV epidemics are still unpredictable and difficult to control (*34*), as confirmed by the surge of cases in 2012, resulting in 286 reported deaths, after years of relatively mild epidemic years (http://www.cdc.gov/westnile/resources/pdfs/data/2012wnvhumaninfectionsbystate.pdf).

Previous studies have noted older age and male sex as predictors of severe outcomes (e.g., NID) (*5*,*35*,*36*), but not for detection of WNV RNA, as observed in our study. The strong association that we found with white race/ethnicity is novel and is likely reflective of the fact that more white than nonwhite persons donate blood.

Using data for WNV NAT–positive donors, we estimated that 555,037 WNV infections occurred in the United States during 2010–2012. During that period, ArboNet reported a total of 7,407 NID and non-NID cases (http://www.cdc.gov/westnile/statsmaps/finalmapsdata/index.html). Busch et al. (*9*) estimated that 735,000 WNV infections occurred during 2003 in the United States, and ArboNet reported 9,862 NID and non-NID cases in 2003 (http://www.cdc.gov/westnile/resources/pdfs/data/2003wnvhumaninfectionsbystate.pdf). Cervantes et al. (*29*) reported an estimated 85,156 WNV infections in northern Texas during the 2012 epidemic, compared with our estimate of 93,004 infections in all of Texas that year.

We report a weighted ratio of 1 NID case per 141 WNV infections during 2010–2012, similar to the ratio of 1 NID case per 140 WNV infections reported during the 1999 New York outbreak (*5*). The difference between our estimate and the estimate reported by Busch et al. (*9*) for the year 2003 (1 NID case/256 WNV infections) may reflect yearly variations of the disease, data quality, or, most probably, the differences in the WNV NAT positivity window used in the study by Busch et al. (6.9 days) and in our study (15.1 days), which could have resulted in an overestimate of WNV incidence and NID ratio in 2003. Since the 1999 outbreak in New York, genetic evolution of WNV has been described or hypothesized in the United States and elsewhere with a hypothetical increase in virus fitness and pathogenicity (*37*–*40*). The virulent lineage 2 WNV has been implicated in increasing epidemics in Europe and Russia and with devastating cases of NID (*41*). Our findings do not support a change in virus penetrance in the United States that might have resulted in the higher number of deaths reported during the 2012 season.

The ratio of infection cases to NID cases is a good surveillance strategy for WNV pathogenic evolution. Although issues with case recognition and passive reporting may result in underreporting of NID cases in the general population, data on NID incidence may be more reflective of the total population that is covered by public health surveillance (*2*,*28*). On the other hand, decreased public health communication during low-incidence epidemic years could result in underreporting of WNV infections, and increased communication during more severe epidemics could result in more complete reporting.

Strengths of the current study include a very large study population spanning a large geographic region of the United States and a uniform blood donor sampling frame and test methods for WNV RNA. Limitations include geographic gaps in participating blood centers, leading to a potentially biased estimate of incidence in certain states. Blood donor incidence detected by NAT may underestimate infection rates in the general population by as much as 25% because of self-exclusion from donation due to WNV signs and symptoms (*42*), resulting in self-selected healthy donors. Also, blood collection centers do not draw from entire states, so some areas are proportionally not represented (*29*). Because we used operational data, we had only a limited number of demographic variables and no information on potential exposures to WNV. Last, because we did not have data on whether MP-NAT or ID-NAT screening was used for each donation, we used an average RNA detection duration period of 15.1 days and, thus, may have slightly underestimated or overestimated WNV incidence (*29*).

In conclusion, we used a large nationwide dataset obtained from a consortium of blood collection organizations to strengthen the idea that monitoring US blood donations for WNV RNA is a useful surveillance tool for studying the evolution of epidemics and potentially associated pathogenicity. WNV RNA blood donation data are useful for tracking epidemics prospectively (because they are collected in real time) and retrospectively as a complement to existing case-based WNV surveillance networks in the United States.

Technical Appendix Estimated West Nile Virus infection and neuroinvasive disease incidences, by year and state, USA, 2010–2012.
