# Supplementary material for: Correlation of West Nile Virus Incidence in Donated Blood with West Nile Neuroinvasive Disease Rates, United States, 2010–2012
Source: Emerg Infect Dis. 2017 Feb;23(2):212–9. doi: 10.3201/eid2302.161058 (PMC5324803; doi:10.3201/eid2302.161058)
Supplement: Technical Appendix — Estimated West Nile Virus infection and neuroinvasive disease incidences, by year and state, USA, 2010–2012. [file 16-1058-Techapp-s1.pdf]

Article DOI: http://dx.doi.org/10.3201/eid2302.161058

Correlation of West Nile Virus Incidence in Donated Blood with West Nile Neuroinvasive Disease Rates, United States, 2010–2012

Technical Appendix

Technical Appendix Table 1. Incidence estimation and some related measures by state during 2010 season for the entire United States

State	State population 2010	Donations 5-mo period	WNV NAT positives 5-mo period	Estimated incidence (95% CI)	Estimated WNV infections 5-mo period	Seasonal NID	Inverse ratio NID/WNV (95% CI)
Alaska	714,046	213	0	0	0	0	
Alabama	4,784,762	42,806	0	0	0	1	
Arkansas	2,922,750	39,348	0	0	0	6	
Arizona	6,410,810	101,120	33	329 (293–364)	21,081	107	197
California	37,334,410	261,385	1	4 (0–8)	1,420	72	20
Colorado	5,048,472	4,274	0	0	0	26	
Connecticut	3,576,616	50,748	1	19 (10–27)	684	7	98
District of Columbia	604,989	3,829	0	0	0	3	
Delaware	899,824	1,101	1	777 (723–834)	6,997	0	
Florida	18,845,967	163,422	1	6 (1–11)	1,256	9	140
Georgia	9,714,748	117,437	1	8 (2–14)	805	4	201
Hawaii	1,364,274	267	0	0	0	0	
Iowa	3,050,321	22,620	1	41 (29–54)	1,277	5	255
Idaho	1,570,784	32,712	0	0	0	0	
Illinois	12,840,459	81,032	1	12 (5–19)	1,610	45	36
Indiana	6,489,856	68,283	0	0	0	6	
Kansas	2,858,837	51,446	9	171 (146–197)	4,906	4	1,227
Kentucky	4,346,655	40,616	4	96 (77–115)	4,189	2	2,095
Louisiana	4,544,125	22,590	1	47 (34–61)	2178	20	109
Massachusetts	6,563,259	77,791	1	12 (5–19)	812	6	135
Maryland	5,787,998	83,494	1	12 (5–19)	700	17	41
Maine	1,327,585	29,613	0	0	0	0	
Michigan	9,877,670	161,254	2	12 (5–19)	1,207		48
Minnesota	5,310,737	91,005	0	0	0	4	
Missouri	5,996,092	71,772	0	0	0	3	
Mississippi	2,969,137	25,920	0	0	0	3	
Montana	990,735	26,046	0	0	0	0	
North Carolina	9,559,048	167,132	2	11 (5–18)	1108	0	
North Dakota	674,363	20,889	0	0	0	2	
Nebraska	1,829,696	56,073	16	285 (252–318)	5,225	10	522
New Hampshire	1,316,843	29,544	0	0	0	1	
New Jersey	8,803,388	77,136	4	52 (38–66)	4,585	15	306
New Mexico	2,064,767	31,803	6	187 (161–215)	3,879	21	185
Nevada	2,703,758	39,713	0	0	0	0	
New York	19,399,242	283,624	19	70 (54–86)	13,607	89	153
Ohio	11,538,290	217,890	2	9 (3–15)	1,061	4	265
Oklahoma	3,759,482	20,503	0	0	0	1	
Oregon	3,838,212	84,557	0	0	0	0	
Pennsylvania	12,711,308	155,693	6	38 (26–51)	4,889	19	257
Rhodes Island	1,052,769	672	0	0	0	0	
South Carolina	4,635,835	54,988	0	0	0	1	
South Dakota	816,223	19,740	0	0	0	4	
Tennessee	6,356,673	40,046	0	0	0	2	
Texas	25,242,683	196,098	12	62 (47–78)	15,747	77	205

State	State population 2010	Donations 5-mo period	WNV NAT positives 5-mo period	Estimated incidence (95% CI)	Estimated WNV infections 5-mo period	Seasonal NID	Inverse ratio NID/WNV (95% CI)
Utah	2,775,093	49,582	1	18 (10–27)	520	1	520
Virginia	8,025,105	86,816	4	46 (33–59)	3,705	4	926
Vermont	625,916	18,719	0	0	0	0	
Washington	6,743,636	41,404	0	0	0	1	
Wisconsin	5,689,591	69,621	0	0	0	0	
West Virginia	1,854,019	24,842	0	0	0	0	
Wyoming	564,367	11,176	0	0	0	2	
Total	309,326,225	3,470,405	130	33.4 (22–45)	103,450	629	164 (152–178)

Technical Appendix Table 2. Incidence estimation and some related measures by state during 2011 season for the entire United States

State	State population 2011	Donations 5-mo period	WNV NAT positives 5-mo period	Estimated incidence (95% CI)	Estimated WNV infections 5-mo period	Seasonal NID	Inverse ratio NID/WNV (95% CI)
Alaska	723,860	850	0	0	0	-	
Alabama	4,803,689	37,550	0	0	0	5	
Arkansas	2,938,582	30,166	0	0	0	1	
Arizona	6,467,315	99,574	16	162 (138–188)	10,524	49	215
California	37,683,933	255,833	10	38 (26–50)	14,423	110	131
Colorado	5,116,302	4,955	0	0	0	2	
Connecticut	3,586,717	50,991	0	0	0	8	
District of Columbia	619,020	3,935	2	556 (510–602)	3,441	10	344
Delaware	908,137	961	0	0	0	1	
Florida	19,082,262	162,409	2	13 (6–20)	2,507	20	125
Georgia	9,812,460	113,223	4	36 (24–48)	3,550	14	254
Hawaii	1,378,129	443	0	0	0	-	
Iowa	3,064,097	22,659	0	0	0	5	
Idaho	1,583,744	31,570	0	0	0	1	
Illinois	12,859,752	78,459	1	12 (6–20)	1,654	22	75
Indiana	6,516,353	68,134	2	30 (32–59)	2,000	7	286
Kansas	2,870,386	46,606	0	0	0	4	
Kentucky	4,366,814	38,971	2	45 (32–59)	1,991	4	498
Louisiana	4,574,766	24,903	1	44 (32–58)	2,047	6	341
Massachusetts	6,607,003	76,868	3	38 (26–51)	2,557	5	511
Maryland	5,839,572	78,522	5	64(48–80)	3,750	10	375
Maine	1,328,544	33,815	0	0	0	-	
Michigan	9,876,801	155,907	1	6 (1–11)	637	32	20
Minnesota	5,347,299	87,929	0	0	0	1	
Missouri	6,008,984	66,711	0	0	0	6	
Mississippi	2,977,457	23,881	0	0	0	31	
Montana	997,667	27,283	0	0	0	1	
North Carolina	9,651,103	158,872	0	0	0	2	
North Dakota	684,740	21,102	0	0	0	1	
Nebraska	1,842,234	56,178	6	104 (85–125)	1,928	14	138
New Hampshire	1,317,807	30,406	0	0	0	-	
New Jersey	8,834,773	68,976	0	0	0	2	
New Mexico	2,078,674	30,150	2	69 (53–86)	1,447	4	362
Nevada	2,720,028	43,130	2	48 (35–62)	1,322	12	110
New York	19,501,616	282,719	7	26 (16–36)	5,163	28	184
Ohio	11,541,007	213,987	9	42 (29–55)	4,874	10	487
Oklahoma	3,784,163	18,783	0	0	0	1	
Oregon	3,868,229	73,652	0	0	0	-	
Pennsylvania	12,743,948	138,552	1	6 (2–12)	888	5	178
Rhodes Island	1,050,646	1,791	0	0	0	1	
South Carolina	4,673,348	52,744	0	0	0	-	
South Dakota	823,593	19,977	0	0	0	-	
Tennessee	6,399,787	41,704	1	26 (16–36)	1,681	16	105
Texas	25,631,778	193,121	4	20 (12–29)	5,295	20	265
Utah	2,814,347	48,308	1	18 (10–27)	533	1	533
Virginia	8,104,384	82,723	3	34 (23–46)	2,834	8	354
Vermont	626,592	19,259	1	47 (34–61)	299	1	299
Washington	6,823,267	37,011	0	0	0	-	
Wisconsin	5,709,843	68,923	0	0	0	2	
West Virginia	1,854,908	23,975	2	87 (69–106)	1,627	2	813
Wyoming	567,356	11,292	0	0	0	1	

State	State population 2011	Donations 5-mo period	WNV NAT positives 5-mo period	Estimated incidence (95% CI)	Estimated WNV infections 5-mo period	Seasonal NID	Inverse ratio NID/WNV (95% CI)
Total	311,587,816	3,360,443	88	24.7 (15–34)	76,975	486	158 (145–174)

Technical Appendix Table 3. Incidence estimation and some related measures by state during 2012 season for the entire United States

State	2012 state population	Donations 5-mo period	WNV NAT positives 5-mo period	Estimated incidence (95% CI)	Estimated WNV infections 5-mo period	Seasonal NID	Inverse ratio NID/WNV (95% CI)
Alaska	731,449	153	0	0	0	0	
Alabama	4,822,023	38,174	14	371 (333–409)	17,910	38	471
Arkansas	2,949,131	30,243	1	36 (24–48)	1,068	44	24
Arizona	6,553,255	101,093	34	337 (301–375)	22,118	87	254
California	38,041,430	259,553	20	79 (61–97)	30,223	297	102
Colorado	5,187,582	3,726	0	0	0	62	
Connecticut	3,590,347	56,898	1	17 (9–26)	631	12	53
District of Columbia	632,323	3,344	0	0	0	8	
Delaware	917,092	934	0	0	0	2	
Florida	19,317,568	162,140	7	43, (30–56)	8,345	52	160
Georgia	9,919,945	114,676	13	115 (94–136)	11,464	46	249
Hawaii	1,392,313	165	0	0	0	0	
Iowa	3,074,186	22,256	2	110 (90–131)	3,409	11	310
Idaho	1,595,728	32,095	3	98 (79–118)	1,573	5	315
Illinois	12,875,255	78,151	5	62 (47–78)	8,071	187	43
Indiana	6,537,334	68,067	10	149 (126–174)	9,791	46	213
Kansas	2,885,905	47,800	21	452 (410–494)	13,051	20	653
Kentucky	4,380,415	41,478	1	23 (14–32)	1,013	13	78
Louisiana	4,601,893	22,576	1	47 (34–61)	2,182	155	14
Massachusetts	6,646,144	76,648	2	26 (16–36)	1,746	25	70
Maryland	5,884,563	71,657	17	244 (214–275)	14,390	25	576
Maine	1,329,192	31,059	0	0	0	1	
Michigan	9,883,360	136,046	21	147 (123–171)	14,529	141	103
Minnesota	5,379,139	90,293	28	312 (278–347)	16,824	34	495
Missouri	6,021,988	71,375	4	57 (43–72)	3,472	17	204
Mississippi	2,984,926	24,322	6	262 (230–294)	7,823	103	76
Montana	1,005,141	25,751	0	0	0	1	
North Carolina	9,752,073	152,024	3	21 (12–31)	2,111	7	302
North Dakota	699,628	20,233	16	766 (713–821)	5,366	39	138
Nebraska	1,855,525	57,869	32	570 (523–617)	10,577	42	252
New Hampshire	1,320,718	29,393	0	0	0	1	
New Jersey	8,864,590	70,621	4	55 (41–70)	4,928	22	224
New Mexico	2,085,538	29,967	6	207 (179–235)	4,320	24	180
Nevada	2,758,931	38,117	1	26 (16–36)	720	5	144
New York	19,570,261	268,489	10	38 (26–50)	7,443	61	122
Ohio	11,544,225	172,631	18	97 (78–116)	11,207	76	147
Oklahoma	3,814,820	17,497	6	362 (326–400)	13,846	103	134
Oregon	3,899,353	78,319	0	0	0	0	
Pennsylvania	12,763,536	134,780	9	67 (52–84)	8,673	33	263
Rhodes Island	1,050,292	765	0	0	0	2	
South Carolina	4,723,723	53,498	6	112 (91–133)	5,300	20	265
South Dakota	833,354	18,476	26	1,465 (1390–1550)	12,213	62	197
Tennessee	6,456,243	42,327	0	0	0	19	
Texas	26,059,203	191,501	65	356 (320–394)	93,004	844	110
Utah	2,855,287	48,583	0	0	0	3	
Virginia	8,185,867	76,524	1	12 (6–20)	1,060	20	53
Vermont	626,011	18,971	0	0	0	1	
Washington	6,897,012	39,714	0	0	0	4	
Wisconsin	5,726,398	72,314	5	76 (59–94)	4,386	44	100
West Virginia	1,855,413	22,384	1	41 (29–54)	772	5	154
Wyoming	576,412	11,335	2	183 (157–210)	1,056	3	352
Total	313,914,040	3,277,005	422	119.9 (98–141)	376,612	2,872	131 (126–136)
